# Texture tomography with high angular resolution utilizing sparsity

**DOI:** 10.1107/S1600576725001426

**Published:** 2025-03-13

**Authors:** Mads Carlsen, Florencia Malamud, Peter Modregger, Anna Wildeis, Markus Hartmann, Robert Brandt, Andreas Menzel, Marianne Liebi

**Affiliations:** a Paul Scherrer Institut 5232 Villigen PSI Switzerland; b Physics Department University of Siegen 57072Siegen Germany; c Center for X-ray and Nano Science CXNS Deutsches Elektronen-Synchrotron DESY 22607Hamburg Germany; d Mechanical Engineering Department University of Siegen 57072Siegen Germany; e Institute of Materials, Ecole Polytechnique Fédérale de Lausanne,1015Lausanne, Switzerland; f Department of Physics Chalmers University of Technology SE-412 96Gothenburg Sweden; Montanuniversität Leoben, Austria

**Keywords:** tomography, texture analysis, XRD-CT, X-ray diffraction computed tomography, 3D-XRD, 3D X-ray diffraction

## Abstract

We demonstrate an approach to the reconstruction of scanning probe X-ray diffraction tomography data with anisotropic polycrystalline samples. The method involves reconstructing a voxel map containing an orientation distribution function in each voxel of a bulk 3D sample. By choosing a particular kind of basis functions, we can effectively utilize non-negativity in orientation space for samples with sparse texture. This enables us to achieve stable solutions at high angular resolutions where the problem would otherwise be underdetermined. This method differs from established approaches by not relying on a peak-finding step. It is therefore applicable to sample systems consisting of small and highly mosaic crystalline domains that are not handled well by these methods. We demonstrate the new approach using data from a shot-peened martensite sample where we are able to map the twinning microstructure in the interior of a bulk sample without resolving the individual lattice domains. We also demonstrate the approach on a piece of gastropod shell with a mosaic microstructure. The results suggest that, by utilizing the sparsity of the texture, the experiment can be carried out using only a single rotation axis, unlike previous demonstrations of texture and tensor tomography.

## Abstract 2

This article reports non-destructive 3D texture mapping of a wide range of polycrystalline materials using synchrotron X-rays and utilizing the sparsity of the reconstructed texture.

## 1. Introduction

Understanding the texture of crystalline materials is central to a wide range of phenomena in materials science. Electron backscatter diffraction (EBSD) has become the main tool for texture characterization over the past 20 years, but EBSD is a surface imaging technique. It cannot see the local environment hidden in the third dimension and its analysis is susceptible to bias by 2D effects (Juul Jensen & Zhang, 2020 ▸; Knipschildt *et al.*, 2023 ▸). While full 3D maps can be obtained by serial sectioning, such imaging is inherently destructive and precludes *in situ* characterization. Therefore, X-ray diffraction techniques play an irreplaceable role in the characterization of microstructure whenever bulk properties are of interest. While traditional X-ray diffraction techniques probe the volume-averaged structure of bulk samples, a range of tomographic techniques have been developed that yield spatially resolved information.

**Figure 1:**
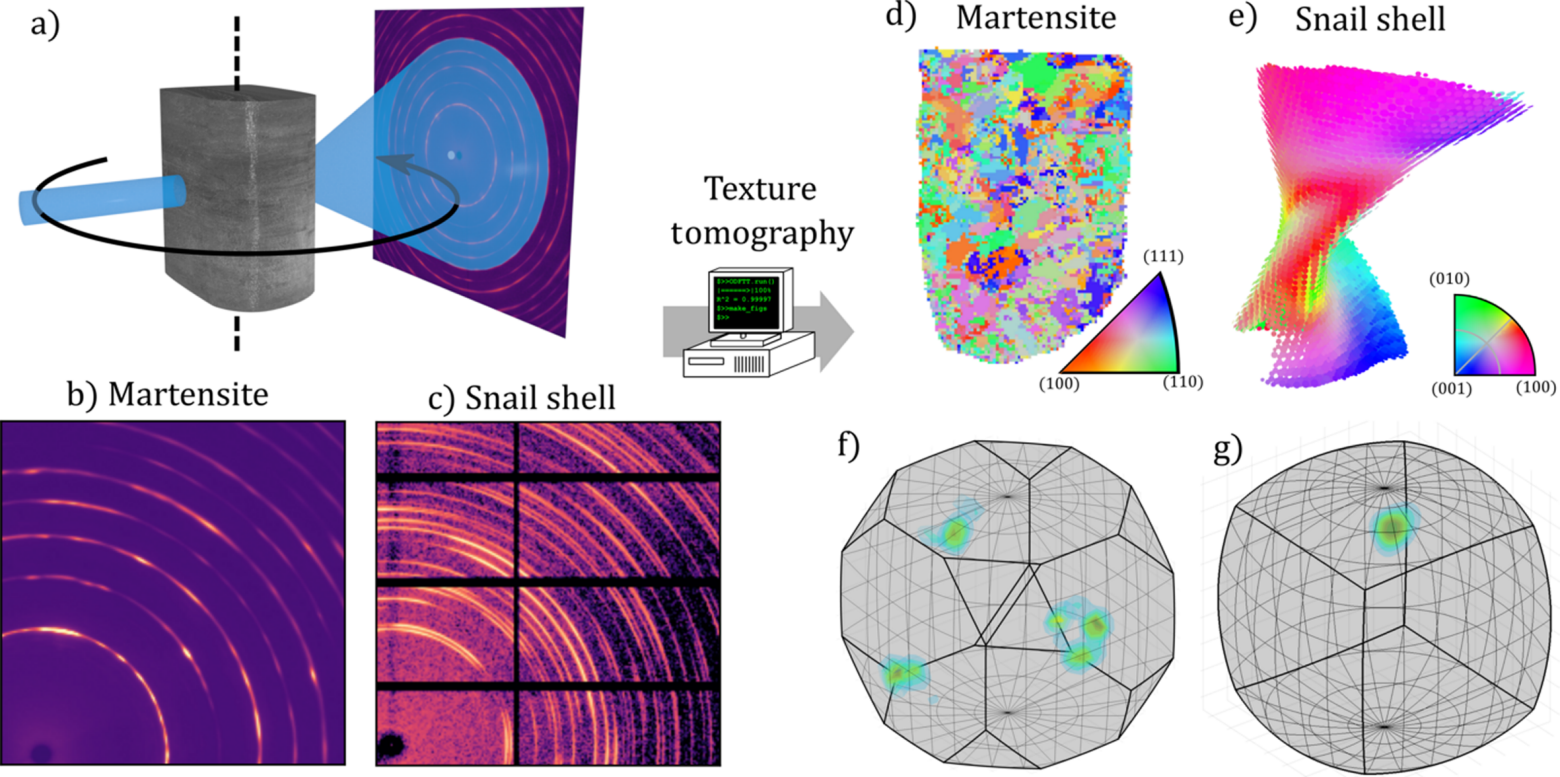
(*a*) Sketch of the experimental geometry and (*b*, *c*) examples of X-ray diffraction patterns from each of the two investigated samples. (*d*, *e*) show the reconstructed samples as inverse pole figure maps of the main orientation and (*f*, *g*) show the full ODFs of a single voxel plotted as a 3D density in Rodriguez vector space in the asymmetric zone of the (*f*) cubic and (*g*) orthorhombic crystal system of ferrite and aragonite, respectively.

In this paper, we present a new approach for analyzing the data from X-ray diffraction computed tomography (XRD-CT) experiments (Harding *et al.*, 1987 ▸; Stock *et al.*, 2008 ▸; Bleuet *et al.*, 2008 ▸). The experiment works by scanning a sample through a point-focused X-ray beam, usually at a synchrotron facility, and rotating the sample to record an X-ray diffraction pattern at each point of a sinogram. The geometry of the experiment is sketched in Fig. 1 ▸(*a*). While traditional XRD-CT assumes an isotropically scattering sample, the technique proposed in this paper explicitly reconstructs the anisotropy of the sample, resulting in a spatially resolved 3D map of the crystallographic texture of a polycrystalline sample.

The proposed method builds on the principles of tensor tomography (Malecki *et al.*, 2014 ▸; Liebi *et al.*, 2015 ▸; Schaff *et al.*, 2015 ▸) and is formally identical to the method developed by Frewein *et al.* (2024 ▸) called texture tomography, except with a different choice of basis functions. This choice of basis functions enables our method to effectively enforce the prior knowledge of sparsity on the solution which is necessary to ensure unique solutions at higher angular resolution (around 10° to 5° in this paper). As texture tomography and the method proposed in this paper reconstruct the orientation distribution function (ODF) in each voxel while the established tensor tomography techniques reconstruct pole figures, we introduce the abbreviations ODF-TT and PF-TT to distinguish between them.

Generally, the proposed method falls within a large family of X-ray techniques which are collectively referred to as 3D X-ray diffraction (3D-XRD) (Poulsen, 2004 ▸), comprising scanning 3D-XRD (s3D-XRD) (Hayashi *et al.*, 2015 ▸), which uses an identical experimental set-up, as well as a number of full-field techniques (Poulsen *et al.*, 2001 ▸; Suter *et al.*, 2006 ▸; Schmidt *et al.*, 2008 ▸; Ludwig *et al.*, 2008 ▸; Vigano *et al.*, 2016 ▸; Oddershede *et al.*, 2019 ▸) all aimed at reconstructing the 3D microstructure of polycrystalline materials from X-ray dif­frac­tion measurements. 3D-XRD techniques rely on spatially resolving the smallest coherent lattice domains of a sample (such as grains, sub-grains or twin domains) in order to index distinct diffraction spots that can be assigned to individual grains. Instead, the tensor and texture tomography methods work with smeared-out Debye–Scherrer rings with a continuous intensity variation along the detector azimuth [as shown in Figs. 1 ▸(*b*) and 1 ▸(*c*)] and reconstruct the spatially averaged texture over multiple crystalline domains. Therefore, they are well suited for small-grained and highly deformed materials such as bone-apatite (Grünewald *et al.*, 2020 ▸; Grünewald *et al.*, 2023 ▸) and cold-worked metals (Carlsen *et al.*, 2024 ▸).

ODF-TT overcomes some of the weaknesses of other X-ray scattering tomography techniques and broadens the range of samples that can be studied. By reconstructing the full ODF using a grid-based expansion of the ODF, non-negativity in orientation space can be enforced, overcoming certain ambiguities of the inversion problem. Notably, because ODF-TT is spatially resolved, only the texture on the length scale of a reconstructed voxel needs to be sparse to make use of the non-negativity constraint and not the texture of the full sample. Furthermore, by enforcing the lattice symmetries we overcome the ‘missing-wedge’ problem of PF-TT (Schaff *et al.*, 2015 ▸; Nielsen *et al.*, 2024 ▸), allowing experiments to be carried out in a simpler geometry without a second rotation stage which is commonly used in PF-TT. We present the mathematical approach to the reconstruction and apply the method to a 2D XRD-CT dataset from a piece of shot-peened martensite and to a full 3D tensor tomography dataset of a piece of gastropod shell.

## 2. Method

In this section we introduce some concepts from texture inversion and show how they can be combined with the methodology of tensor tomography. Finally we discuss how the output of the texture tomography reconstruction is analyzed to extract some meaningful quantities used for visualization.

### 2.1. Texture inversion

While tensor tomography was originally applied for small-angle X-ray scattering (Liebi *et al.*, 2015 ▸; Schaff *et al.*, 2015 ▸) (SAS-TT), its extension to wide-angle Bragg scattering is straightforward (Grünewald *et al.*, 2020 ▸; Carlsen *et al.*, 2024 ▸). In SAS-TT, the reconstructed quantity is typically called the reciprocal-space map. For crystalline systems that only display scattering in discrete shells corresponding to the length of reciprocal-lattice vectors, the reciprocal-space map is more easily represented by maps of the intensity on these spheres only. Such a map, when appropriately normalized, is called a pole figure. These pole figures follow symmetries corresponding to the rotational symmetries of the crystal lattice. However, the symmetries are not fully realized in the individual pole figures but result in correlations between different pole figures. This motivates reconstruction of the ODF which describes the anisotropy of all Bragg peaks simultaneously and allows the rotational symmetries to be enforced more easily on the reconstruction.

The implementation of texture tomography by Frewein *et al.* (2024 ▸) relies on expanding the ODF in a series of symmetrized generalized spherical harmonics (Roe, 1965 ▸; Bunge, 1969 ▸). We instead use a technique that expands the ODF into a series of localized functions placed on a grid of orientations mapping out the asymmetric zone of orientation space under the point-group symmetries of the crystal lattice. Such local functions have been used extensively in texture inversion and are typically (i) the indicator functions of some partition of the asymmetric zone, resulting in a piecewise-constant approximation of the ODF (Ruer & Baro, 1977 ▸; Schaeben, 1994 ▸), (ii) finite elements, which give a piecewise-linear approximation (Barton & Dawson, 2002 ▸) or (iii) spherically symmetric standard functions (Schaeben, 1996 ▸), such as the spherical Gaussian function used in this work.

This change of basis functions has been shown to help overcome the ambiguities of the texture inversion problem by enforcing certain kinds of prior knowledge, allowing one to compute regularized solutions (Schaeben, 1988 ▸). Particularly relevant for this study, it allows non-negativity to be imposed in orientation space as a simple non-negativity constraint on the model coefficients. For sparse textures, where the ODF is close to or equal to zero in extended regions of orientation space, the non-negativity constraint has been observed to resolve the inherent ambiguity of the texture inversion problem (Matthies, 1972 ▸; Dahms & Bunge, 1988 ▸).

### 2.2. The tomographic inversion problem

To write the full forward model, we expand the ODF of the voxel at position *x*, *y*, *z* as a series expansion of ODF basis functions: 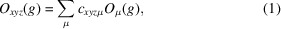
where 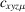 are the unknown expansion coefficients, *g* is a proper rotation and 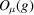 are the basis functions. The basis functions used here are sets of Gaussian radial functions obeying the lattice symmetry rotated to a set of grid points 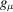 chosen to uniformly fill the asymmetric zone of the respective lattice groups. The ODF defined by this expression is not properly normalized, and the reconstructed quantity is thought of as the density of the crystalline phase times the ODF.

For each ODF basis function, we can pre-compute the corresponding pole figure values at the points probed by the geometry of the experiment. These values are stored in a set of matrices with elements
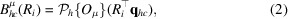
where 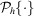 is the pole figure projection operator in ODF space, 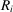 denotes the setting of the rotation stage, and 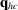 is the scattering vector measured by the detector segment at the azimuthal channels labeled by *c* and the Bragg peak labeled by *h*. These matrix coefficients can be computed with standard approaches from texture inversion and open-source software packages (Hielscher & Schaeben, 2008 ▸).

The full tomographic problem can now be written as 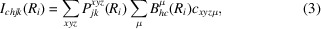
where 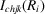 is the five-dimensional dataset containing the integrated intensities and 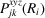 are the matrix elements of the discrete X-ray transform that transforms the voxel grid (*x*, *y*, *z*) to the coordinates of the raster-scan grid (*j*, *k*). In a 2D geometry, the indices *y* and *k* can be omitted and *P* is the Radon transform. To write the problem in this form, we have assumed that the scaling factors which describe the relative intensity of different Bragg peaks are known beforehand and have been normalized out of the measured intensities.

We rewrite the model with linear algebra syntax as 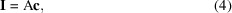
by flattening the dataset into a single vector 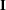, the expansion coefficients into a vector 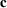, and the products of 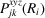 and 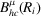 into a single matrix 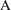. This forms a large system of linear equations that will typically be underdetermined when the ODF expansion is performed to high angular resolution. Here, we will focus on textured materials where the ODF is close to zero in large regions of orientation space, which adds the prior knowledge needed to overcome the inherent ambiguities of the inversion problem. To find the solution, we minimize the square residual with L1 regularization:
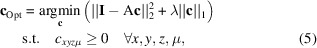
where λ is a regularization parameter and 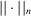 denotes the Euclidean- and 1-norm for *n* = 2 and 1, respectively. To solve this optimization problem, we use projected gradient descent with Nesterov momentum. For both samples investigated in this paper, ODF-TT achieves good reconstructions without regularization, but L1-norm regularization is used to reduce streaking artifacts appearing in the gastropod shell sample (see Appendix *A*[App appa]).

### 2.3. Experiment

#### 2.3.1. Shot-peened martensite

The first sample is a piece of martensitic steel with a cross section of about 0.8 mm by 1 mm. The sample was shot-peened from three sides in order to introduce high residual stresses and varying crystallite sizes from the edges to the center.

The experiment was carried out at the P21.2 beamline (Hegedüs *et al.*, 2019 ▸) of PETRA III (Hamburg, Germany). A photon energy of 68 keV was selected by a double Laue monochromator. Compound refractive lenses shaped a pencil beam of 8 µm width by 3 µm height with a photon flux of about 10^10^ photons s^−1^. A VAREX XRD4343CT detector with a pixel size of 150 µm was positioned about 0.8 m downstream of the sample and provided diffraction angles of up to 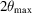 = 14°.

The single rotation axis was oriented in the vertical direction. The sample was scanned with stepwise translations orthogonal to the rotation axis and continuous rotation. In total, 200 projections were collected over 360° and the sample was translated 200 times in 8 µm steps, which resulted in a field of view of 1.6 mm. The exposure time was 0.4 s and a total of 40000 frames were acquired, which took approximately 5 h.

The integrated intensity was determined for each of the seven diffraction rings that were fully captured by the detector and azimuthally regrouped into 72 equally spaced azimuthal bins. The resulting sinograms were corrected by the absorption signal provided by a beamstop diode.

The reconstruction was performed without regularization by running the algorithm for 1000 iterations, which took 8 h to complete. The 2D slice was reconstructed on a 150 × 150 pixel grid with an ODF expansion using 10000 orientations in the asymmetric zone. For each orientation a spherically symmetric Gaussian with σ = 0.05 rad was used. This gives 15 million data points and 240 million degrees of freedom of which 0.6 million were non-zero in the converged solution. The final reconstruction has an 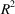 value of 0.8, defined as 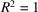
 − 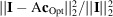.

#### 2.3.2. Gastropod shell

The second sample consists of a piece of the shell of *Helix pomatia* (Roman snail) which was found in the forest at Beznau, Aargau, Switzerland. A small piece measuring 3 mm by 3 mm by 2 mm of the columella was taken about 5 mm below the apex [marked with a purple outline in Fig. 4(*b*)]. The snail shell consists mainly of aragonite and no other crystalline components could be identified in the diffraction patterns.

The sample was measured at the cSAXS beamline at the Swiss Light Source (SLS). The experiments were performed with a photon energy of 18 keV and a sample-to-detector distance of 0.2 m using a Pilatus 2M detector. The beam was focused to a spot of approximately 50 × 50 µm and the sample was raster-scanned in two dimensions through the focused beam with continuous translations in one direction and a step size of 50 µm and step scanning with two orthogonal rotation stages. The intensity of the direct beam was measured by the image detector behind a semi-transparent beamstop constructed of several small discs of single-crystal silicon glued together.

For this sample, a full tensor tomography dataset was obtained, containing measurements made at tilt angles extending to 45°. This allows the texture to be reconstructed with the more established PF-TT approach and the two approaches to be compared; it also allows a comparison between using the full dataset and using a zero-tilt geometry only.

The diffraction patterns contain 16 rings that are fully covered by the detector, but due to partial overlaps of some peaks, only eight rings were used for the reconstructions. These rings were azimuthally regrouped into 96 equally spaced bins.

For the reconstructions, we used a grid of 8000 orientations and Gaussian functions with width σ = 0.1 rad for the basis set. The gastropod shell consists of aragonite of the orthorhombic crystal class, which means the rotation group contains only four symmetries in contrast to 24 in the cubic crystal class of ferrite. Therefore, even though the numbers of grid orientations are similar, the angular resolution of the gastropod shell reconstruction is about a factor of two worse than that of the martensite reconstruction. The full 3D problem has 600 million data points with 150 million in the zero-tilt part, and the model uses 1.8 billion degrees of freedom.

### 2.4. Analysis of reconstructions

Each voxel of the reconstruction contains up to about 10000 independent grid orientations and coefficients. While most coefficients are zero, which simplifies the analysis, both visualization and analysis of the reconstruction remain challenging.

The main tool for visualization used in this paper is to select a main orientation for each voxel, which is defined as the orientation corresponding to the basis function with the largest coefficient. With this main orientation field, we can compute inverse pole figure maps and orientations of crystallographic axes similar to what is done in EBSD analysis. The fraction of intensity attributable to this main orientation is computed by first selecting all orientations that fall within a 20° distance of the main orientation. The fraction is then defined as the sum of all selected coefficients divided by the sum of all orientations.

Furthermore, we use an approximate texture index defined as 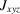 = 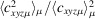. A texture index of one corresponds to a uniform texture and a higher texture index generally corresponds to a sample with stronger texture, increasing towards infinity.

For the martensite sample, the reconstructed texture in most voxels consists of clusters of nearby orientations that have non-zero coefficients corresponding to a texture component with some width larger than the grid resolution. We use a simple approach to find these clusters, where the main orientation is taken to be the orientation with the largest coefficient. Afterwards, orientations within a 20° radius are excluded and a secondary orientation is chosen as the highest coefficient among those remaining. The cutoff distance is needed to avoid classifying two coefficients from the same texture component as separate texture components. A cutoff of 20° is chosen for this sample to avoid finding small misorientations inside the same Bain group, as these are thought to be too close to the grid resolution to allow one to resolve the misorientation axis well by this approach. Better algorithms for clustering of texture components are expected to yield improved results, but the implementation of standard clustering algorithms is not straightforward due to the complicated boundary conditions of orientation space under point-group symmetries.

From these clusters, we compute the misorientation. Calling the main and the secondary orientations 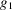 and 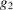, respectively, the misorientation is defined as the orientation 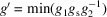 for 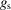 in the symmetry group. The misorientation is then conjugated using 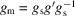 such that the axis of rotation falls within the fundamental region of the inverse pole figure.

For the gastropod shell sample, the maps computed using the main orientation appear noisy. To address this, we compute a mean orientation that also takes into account the weights of neighboring grid orientations. This is done by first selecting all orientations that fall within a 20° radius of the main orientation. From these selected orientations, a mean is computed by first rotating all orientations into a frame centered on the main orientation by a right-handed application of the inverse of the main orientation, making sure to pick the smallest symmetry-equivalent orientation for each. The transformed orientations are afterwards cast to Rodriguez vector space and a weighted arithmetic mean is computed of the vector components using the coefficients as weights. Finally, this mean orientation difference is transformed back into the original frame to give the mean orientation used for the plots.

## 3. Results

To test the feasibility of ODF-based tensor tomography, we show results using two separate experimental datasets. One is a piece of martensitic steel which is a sample system of high scientific interest with a well known twinned microstructure. It has not so far been possible to map this microstructure with 3D-XRD approaches. The other is a biomineral sample where a full 3D tensor tomography dataset is available, which allows us to compare the performance of ODF-TT with PF-TT. Figs. 1 ▸(*b*) and 1 ▸(*c*) show examples of raw detector frames from each of these two datasets. Both datasets display strongly anisotropic Debye–Scherrer rings but lack easily identifiable separate diffraction spots.

**Figure 2:**
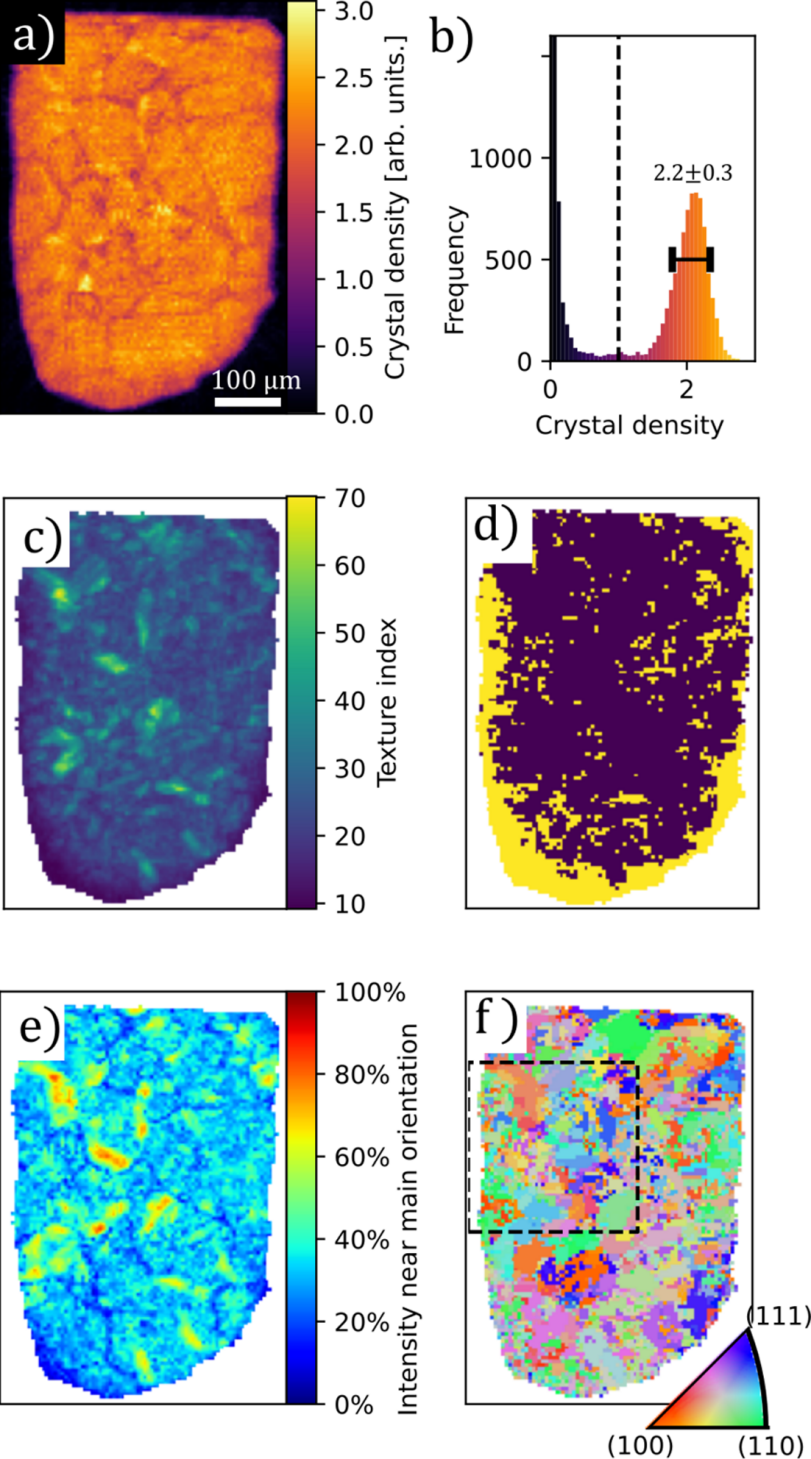
Reconstructed tomogram of the steel sample. (*a*) Crystal density; (*b*) histogram of the reconstructed density values. The dashed line marks the threshold used to generate the mask distinguishing the sample from the air. (*c*) Approximate texture index computed from the standard deviation of the reconstructed coefficients of each pixel. (*d*) A binarized map showing regions with texture index lower than 20 in yellow. (*e*) Fraction of the ODF density that falls within a sphere of radius 20° of the main orientation. (*f*) Inverse pole figure map of the main orientation determined as the grid orientation corresponding to the largest coefficient in each voxel.

Fig. 2 ▸ shows a number of quantities computed from the reconstruction of the martensite dataset. In Figs. 2 ▸(*a*) and 2 ▸(*b*) we see that the outline of the sample is sharp and that the reconstructed density is fairly homogeneous, as expected for the sample which consists almost entirely of ferrite. In Fig. 2 ▸(*c*) we display the texture index of the individual voxels. We observe that the shot-peened surfaces (that is, all surfaces except the straight edge at the top of the figure) display a markedly lower texture index than the rest of the sample. This observation is illustrated in Fig. 2 ▸(*d*) by highlighting voxels with low texture index.

We can determine a main orientation in each voxel. This main orientation is plotted as an inverse pole figure map in Fig. 2 ▸(*f*). The microstructure of martensite consists of narrow laths of ferrite with sub-micrometre thickness which is smaller than the resolution of the experiment. The lattice orientations in the individual laths are related to those of their neighbors due to transformation twinning, which causes specific orientation relationships with neighboring laths and similar orientations to be repeated in nearby crystalline regions. The domains observed in the reconstruction are thus not the individual crystalline domains but rather the outlines of the parent austenite grains that existed before transformation into martensite and high-order subdivisions known as packets. This is demonstrated in Fig. 2 ▸(*e*) which shows that only about half of the total intensity can be attributed to a main orientation. Fig. 2 ▸(*f*) shows the main orientation plotted as an inverse pole figure map. Some large domains up to 50 µm in size are present, but regions with domains of size approaching the resolution of the experiment are also observed.

**Figure 3:**
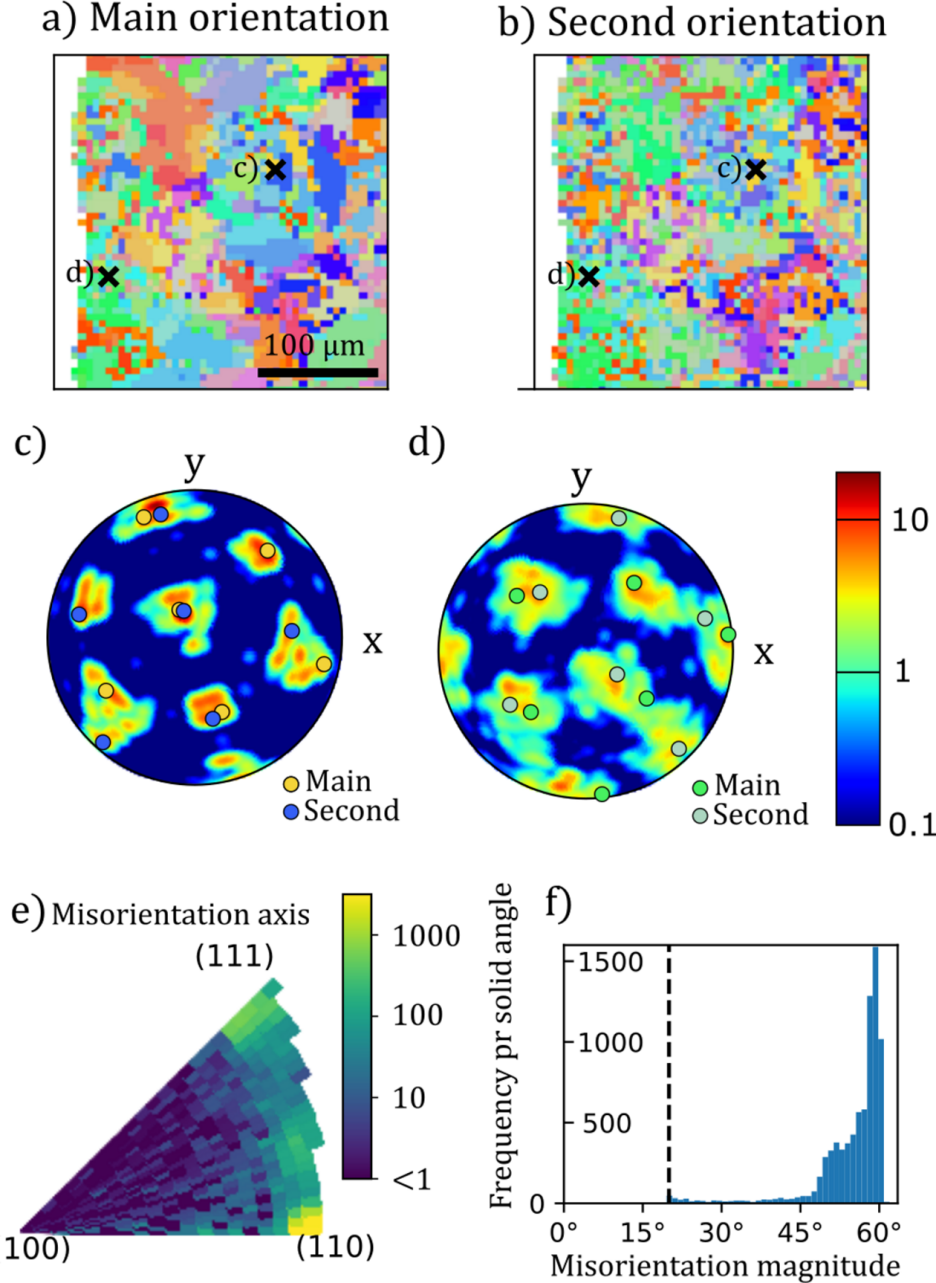
Zoomed-in view of the region of the martensite sample marked in Fig. 2 ▸(*f*). Inverse pole figure maps of the (*a*) primary and (*b*) secondary orientations. (*c*, *d*) show the {110} pole figures of two 4 × 4 pixel regions, marked in (*a*) and (*b*). Overlaid on these pole figures are the poles corresponding to the main orientations colored by the corresponding inverse pole figure color. Histograms of (*e*) the direction of the misorientation axis between the primary and secondary orientations in lattice coordinates and (*f*) the magnitude of the misorientation.

In Fig. 3 ▸ we zoom in and provide a view of the local texture. The texture of fully transformed martensite contains many distinct texture components that fall into three Bain groups, separated by large misorientations (>45°). The smaller misorientations within each Bain group are around 10°, only about two times larger than the grid resolution of the ODF model used for the reconstruction. For select regions of the sample, we see that the pole figures show features typical of the Young–Kurdjumov–Sachs (Young & Smith, 1926 ▸; Kurdjumow & Sachs, 1930 ▸; Guo *et al.*, 2004 ▸) orientation relationship [Fig. 3 ▸(*c*)], while at other points, especially close to the shot-peened edge of the sample, the texture appears less well ordered [Fig. 3 ▸(*d*)].

To quantify the twinning in the whole sample, we compute a histogram of the intra-voxel misorientations. The determination of intra-pixel misorientations precludes observation of misorientations smaller than 20°. This means that misorien­tations corresponding to martensite variants from within the same Bain group are not observed. When determining the misorientations we are only considering the two strongest texture components in each pixel. This potentially suppresses the observation of weaker texture components that might be present in the sample. Fig. 3 ▸(*e*) shows the distribution of the direction of the misorientation axis in the lattice coordinates. We see that the misorientation is mainly around 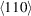 directions and directions close to, but not quite aligned with, 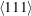. Fig. 3 ▸(*f*) shows the magnitude of the misorientation. The cutoff precludes the observation of misorientations with magnitude smaller than 20°, but beyond this almost no misorientations between 20° and 45° are detected. The found directions and magnitudes of the misorientations are consistent with the expected orientation relationships.

**Figure 4:**
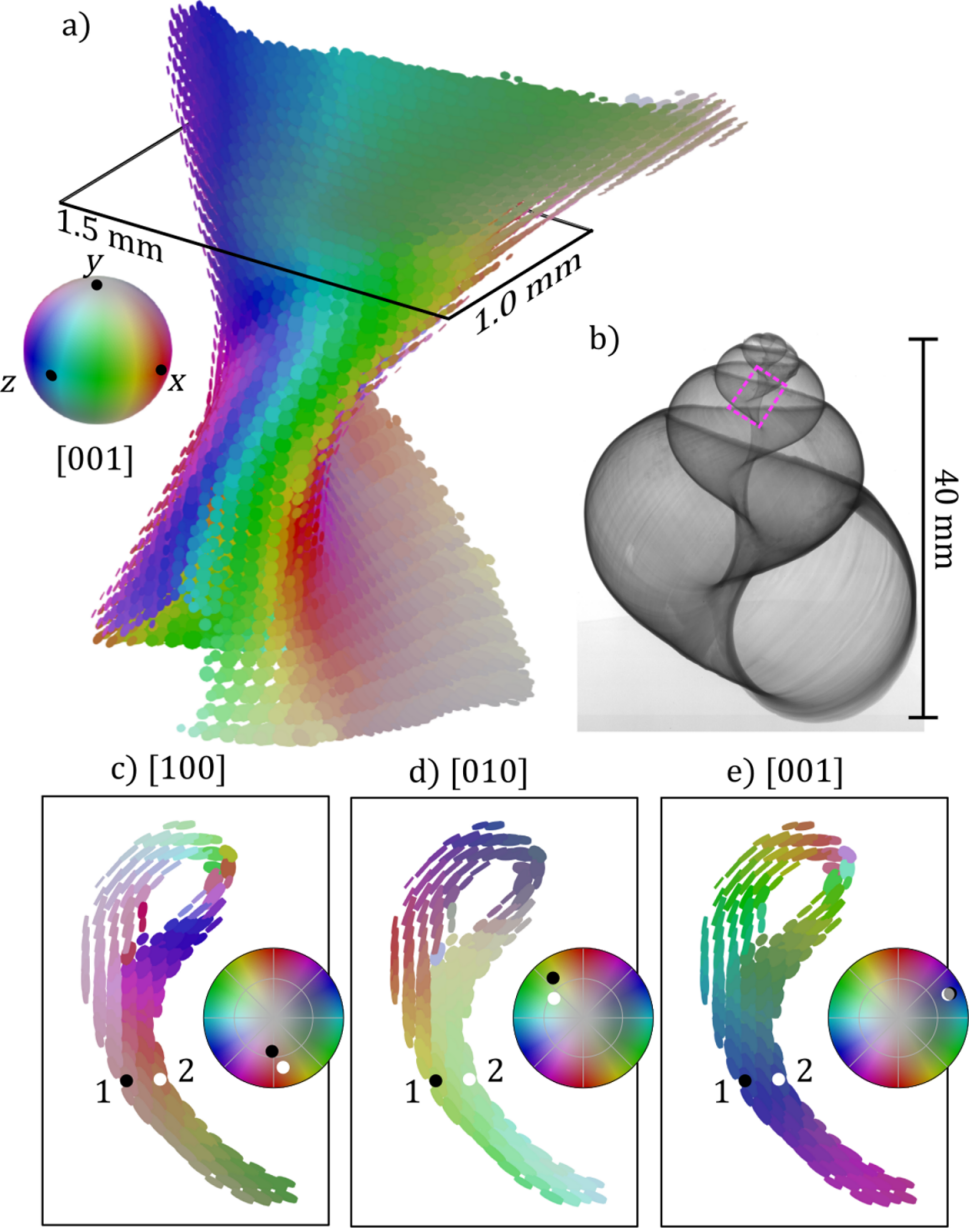
3D renders of the reconstructed snail-shell sample using ODF-TT. (*a*) shows the full reconstructed volume rendered as flat cylinders with the face aligned with the *c* axis of the main orientation and where the size of the cylinders is proportional to the reconstructed density. The cylinders are colored according to the direction of the *c* axis. (*b*) Radiograph of a Roman snail shell showing the approximate location from where the sample was extracted. (*c*, *d*, *e*) Single slice of the reconstructed volume at the location marked with a black rectangle in (*a*) colored according to the direction of the *a*, *b* and *c* axis, respectively. For two select voxels on each side of the columellar wall, labeled 1 and 2, the direction of each of the three primary axes is plotted, showing an abrupt change of about 31° in the direction of the *a* and *b* axes.

The other sample investigated is a piece of gastropod shell and consists predominantly of aragonite. The local microstructure is dominated by a single orientation with a mosaic spread of around 20° FWHM [see Fig. 1 ▸(*g*)]. Fig. 4 ▸(*a*) shows the direction of the orthorhombic *c* axis in the mean orientation. The *c* axis is aligned with the surface normal of the columellar wall, which is typical for aragonite shells in mollusks (Bøggild, 1930 ▸). Figs. 4 ▸(*c*), 4 ▸(*d*) and 4 ▸(*e*) show a single slice of the reconstruction displaying the direction of the three orthorhombic axes. From these figures we can see that where the columellar wall folds back on itself after wrapping around the umbilical it consists of two distinct layers with different crystallographic orientations which are related by a rotation about the common *c* axis. These two layers are part of two distinct whorls of the shell and were grown at different points in the snail’s lifetime.

The gastropod shell dataset can also be reconstructed using established techniques from PF-TT. To give a fair comparison, we use a set of basis functions similar to the ones used in the ODF-TT reconstructions consisting of symmetric Gaussian functions placed on a grid of directions covering the half-sphere (Nielsen *et al.*, 2024 ▸) which can also benefit from the non-negativity constraint and sparse texture. To facilitate comparison between the two reconstruction methods, we chose to reconstruct the 200 Bragg peak as it is the only peak in the dataset that is parallel to one of the main crystallographic axes and has multiplicity 2, which makes it possible to define a main direction of the scattering. The reconstructions were performed using the software package *mumott* (Nielsen *et al.*, 2023 ▸).

**Figure 5:**
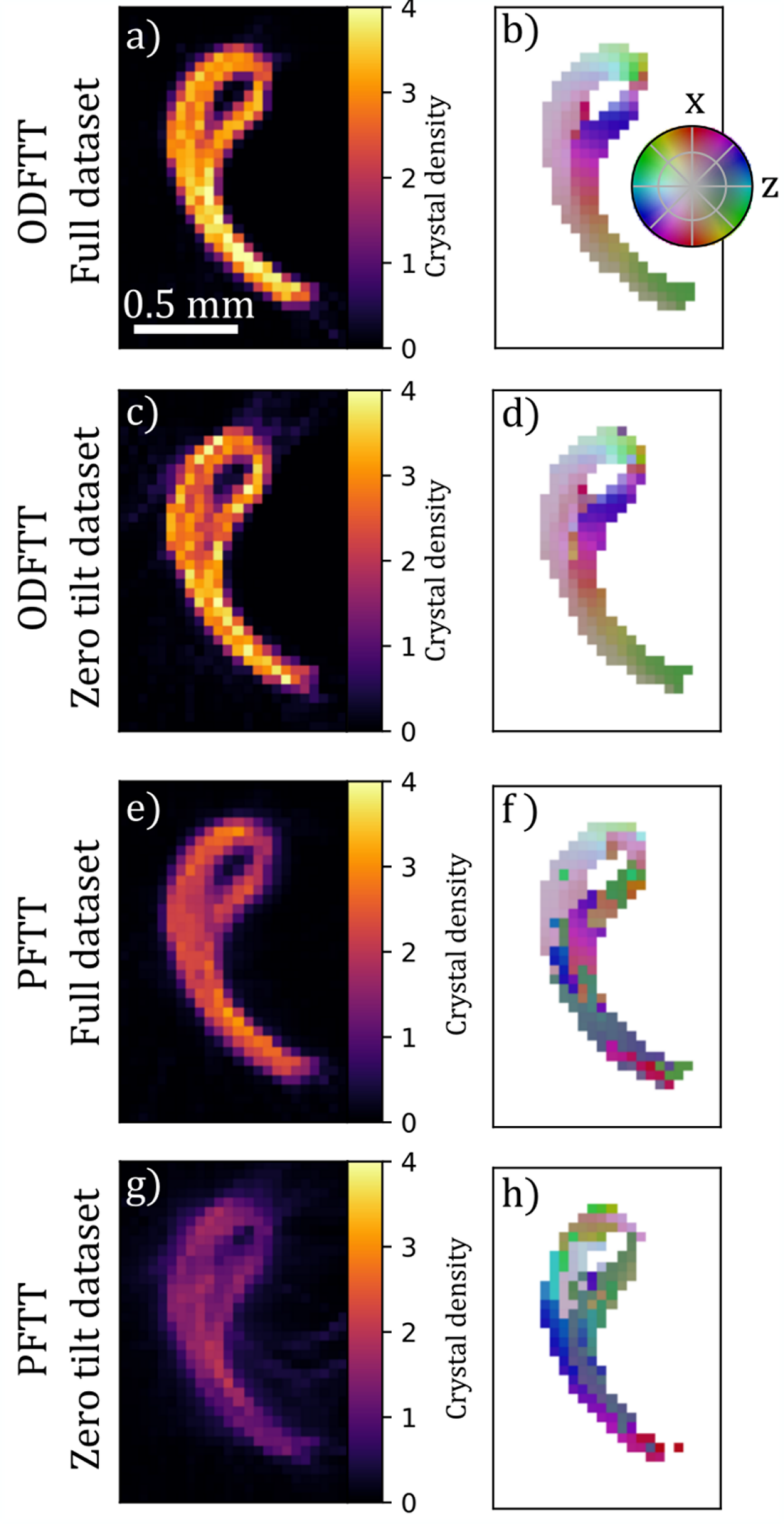
Comparison of reconstructions using ODF-TT and PF-TT of the gastropod shell sample. (*a*, *c*, *e*, *g*) show a single slice, orthogonal to the shell axis, of the reconstructed crystal density while (*b*, *d*, *f*, *h*) show the main orientation of the (100) crystallographic direction using a color coding. (*a*, *b*) are from ODF-TT reconstructions using the full dataset with tilts up to 45° and (*c*, *d*) are from ODF-TT using only the zero-tilt part. (*e*, *f*) are from a PF-TT reconstruction using the full dataset. (*g*, *h*) are from a PF-TT reconstruction made using only the zero-tilt part of the dataset.

**Figure 7:**
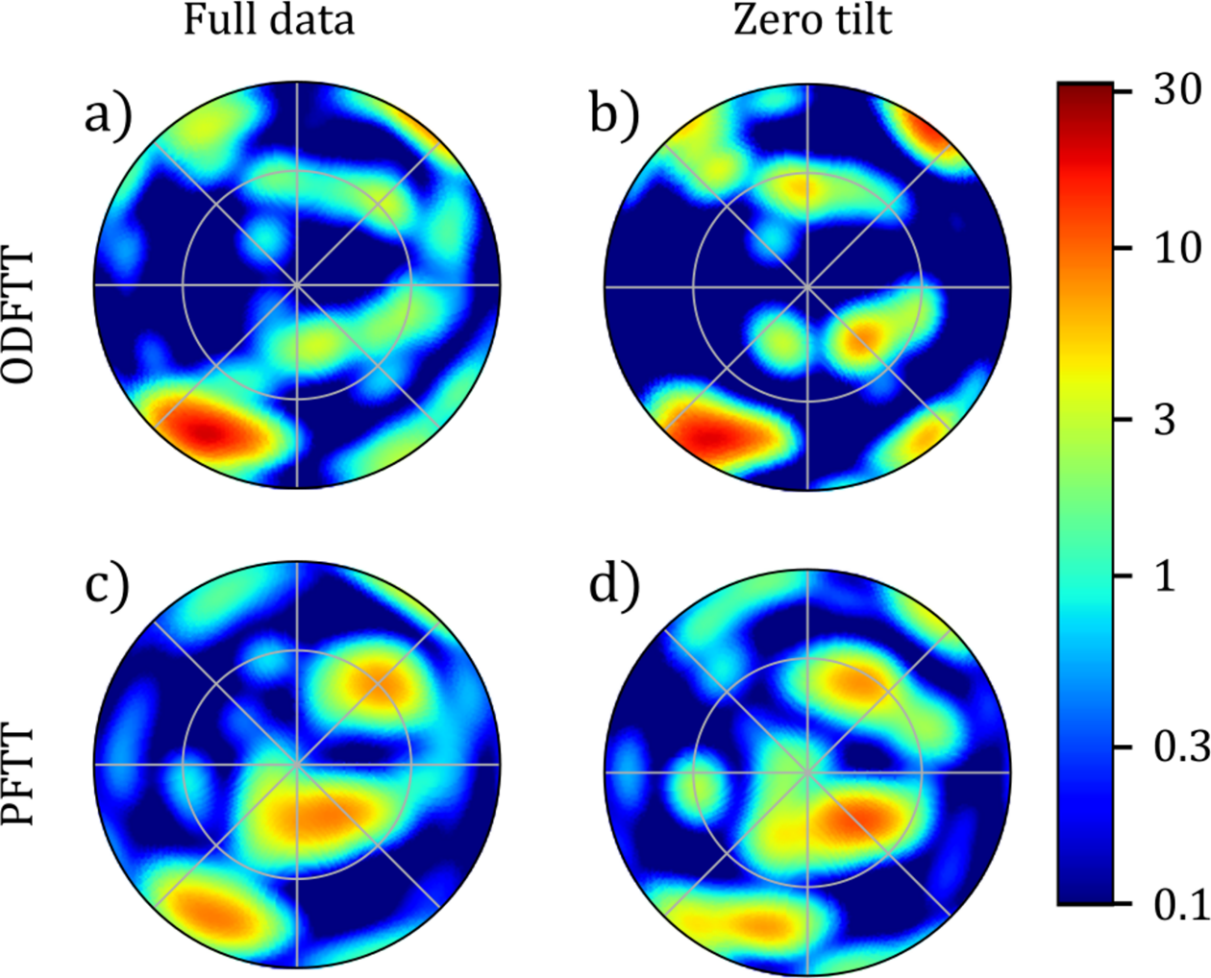
Pole figures of a single voxel of the snail sample with four different reconstructions.

Fig. 5 ▸ shows a comparison between the ODF-TT reconstructions and the PF-TT reconstructions. ODF-TT reconstructs a smoothly varying direction both with the full and zero-tilt datasets except where the columellar wall touches itself after folding around the umbilical. The direction found from the PF-TT reconstructions on the other hand has sudden variations along the columellar wall and appears more erratic. With the full dataset, the PF-TT direction is close to the ODF-TT directions at many points in the sample, especially near the center where the sample is thicker, but differs significantly at the narrower features. This is consistent with the type of ‘missing-wedge’ artifacts common in PF-TT, where flat features are difficult to reconstruct when the projections orthogonal to the normal of the plane have not been measured. With only the zero-tilt data, there are no longer any clear correlations between the direction of the PF-TT reconstruction and that of the ODF-TT reconstructions. Fig. 7 in Appendix *A*[App appa] shows a comparison of individual pole figures, and we see that, while the main peak is also reconstructed by PF-TT, there are additional peaks of similar amplitude that obscure the main orientation.

## 4. Discussion

We have shown that texture tomography can yield well constrained optimization problems, even at high angular resolution where the number of degrees of freedom of the model far exceeds the number of data points, as demonstrated by its application to two distinct samples. The solutions we obtain display the expected textural features of our samples, namely, the Young–Kurdjumov–Sachs orientation relationship in the martensite sample and the orientation of the *c* axis with the surface normal of the snail shell. While these results strongly validate the method, further work is required to fully establish its accuracy and limitations for reconstructing sample texture and shape.

For both samples investigated in this study, the reconstruction problem is underdetermined, with around 240 million and 1.8 billion degrees of freedom for the martensite and snail-shell samples, respectively, in contrast to just 15 million and 0.6 billion data points. Due to the sparsity of the texture however, a large majority of the expansion coefficients are zero in the converged solutions with only 0.6 million and 6 million non-zero coefficients, respectively.

We believe that the sparsity of the voxel-averaged texture is key to providing a well constrained inversion problem. But determining the extent of sparsity needed to provide a well constrained problem is likely to depend on the resolution (in real space and orientation space) of the reconstruction, the point group and the number of measured Bragg peaks, and we do not attempt to answer this question in general. In practice, we have used the fact that the unregularized reconstructions converge to a solution with fewer non-zero coefficients than data points as an indicator that the problem is well constrained.

Further evaluation of the methods to establish the achieved resolution, accuracy and the range of applicability is therefore needed and will require both simulation studies and comparison with other characterization techniques.

The reconstruction problem in pole-figure-based tensor tomography suffers from ambiguities that are due to the experimental difficulty of properly sampling the full range of projection directions. While standard computed tomography problems can be inverted using projections measured around a single rotation axis, PF-TT requires sampling of the full half unit sphere of possible projection directions to be solvable. This necessitates the inclusion of a second rotation stage in the experimental set-up, resulting in longer measurement times. Even with such a sample stage, a range of projection angles are usually obscured by the sample holder, which leads to the missing-wedge problem in PF-TT whereby certain Fourier components of certain scattering directions cannot be probed (Schaff *et al.*, 2015 ▸; Nielsen *et al.*, 2024 ▸).

While it can be shown that full angular sampling is necessary when different scattering directions are reconstructed independently (Schaff *et al.*, 2015 ▸), it is not clear if this is necessary when using a model that enforces correlations between different scattering directions. This has led several authors to assert that PF-TT is possible with such methods (Mürer *et al.*, 2021 ▸; Zhao *et al.*, 2024 ▸) despite systematic studies generally showing this to have a negative impact on the quality of the reconstructions (Liebi *et al.*, 2018 ▸; Carlsen *et al.*, 2024 ▸). As shown here, ODF-TT appears to be able to overcome the missing-wedge problem of PF-TT and achieve solutions without a tilt rotation by utilizing the extra information given by the lattice symmetry and the sparse texture.

A wide range of volumetric grain-mapping techniques using synchrotron X-rays exist. However, they are mostly limited to either materials with low intragranular misorientation and strain or samples with a small number of grains. Recent advances have targeted larger strains by utilizing a focused beam (Hayashi *et al.*, 2015 ▸), conical slits (Hayashi *et al.*, 2023 ▸) or improved computational approaches (Henningsson *et al.*, 2024 ▸). Still, these approaches have so far only been demonstrated on fairly simple grain structures. It has not thus far been possible to reconstruct martensite and other highly strained phases, which Hayashi *et al.* refer to as ‘invisible phases’ (Hayashi & Kimura, 2023 ▸), with 3D-XRD techniques.

Texture tomography differs from these techniques in two key aspects: (i) the measured diffraction patterns are reduced by azimuthal integration rather than by peak finding and (ii) the sample orientation is modeled by an ODF instead of an orientation field.

Because s3D-XRD aims to reconstruct the orientation field, it is necessary for the individual crystalline domains in the sample to be resolved by the resolution of the experiment. In ODF-TT a single voxel can contain multiple texture components which means samples with small crystalline domains can be mapped at a coarse resolution, as long as the assumption of locally sparse texture is maintained. While this can be seen as an advantage, as fine-grained samples can be mapped roughly, allowing faster data collection times, the resulting picture of the sample’s microstructure is less complete, as information such as grain sizes, grain shapes and grain boundary orientations is lost.

If ODF-TT were to be used in a setting where the individual grains are well resolved, the fact that multiple texture components, corresponding to the orientations of neighboring grains, are allowed to be present in the same voxel could lead to lower resolution when compared with s3D-XRD, where each voxel is forced to have only one orientation. Furthermore, because the number of basis functions needed to expand an ODF scales with the cube of the desired resolution, it becomes increasingly slow and computationally expensive to perform ODF-TT at higher resolution. Thus, for materials with well defined grains and sharp peaks in the diffraction patterns, which is the normal setting for 3D-XRD, ODF reconstruction as presented here may not be feasible. However, we still predict that ODF-TT can find applications for coarse-grained metal samples with high plastic deformation, where existing methods struggle due to large overlap of the smeared-out diffraction peaks arising from such deformed grains.

Another advantage of the ODF approach is that the forward model becomes linear, which simplifies the reconstruction and analysis of the problem significantly. This has already been shown and discussed in the 3D-XRD setting by Vigano *et al.* (2014 ▸, 2016 ▸). However, this approach was not applied to the scanning beam geometry.

The output of an ODF-TT reconstruction is an ODF for each voxel in the reconstructed volume, and both analysis and visualization of the reconstruction are challenging. For a sample such as the gastropod shell, where a main orientation can be defined, the information contained in ODFs can be reduced to a small number of derived quantities, such as the orientation field, density and mosaic spread. This is a reduction of the information content of the full reconstruction, as information such as the shape of the texture component and potential existence of secondary texture components is lost.

For samples with more complicated local textures, such as the martensite sample shown here, more detailed analysis is required to interpret the reconstructions. The analysis presented in this paper is a first step and we foresee that more advanced analysis can be developed to extract more information from the reconstructions. This could include processes such as martensite packet and parent grain determination. While methods exist for these tasks, they are typically designed to be used in different settings – with either EBSD images, where the orientation field is well resolved spatially and the task involves grouping neighboring pixels according to their relative misorientations, or traditional X-ray texture mapping which uses a single high-quality texture map of the entire sample. In summary, existing algorithms need to be adapted for in-depth analysis and validation of ODF-TT reconstructions.

## 5. Conclusion

We have demonstrated a novel approach for tomographic 3D mapping of crystallographic texture in bulk samples using a method similar to pole-figure-based tensor tomography. By utilizing grid-based basis functions and leveraging sparsity, we can overcome the inherent ambiguities of the inversion problem. Our proof-of-principle experiments show that a reconstruction can be achieved even for highly under-constrained problems by applying a non-negativity constraint in samples with locally sparse textures.

Unlike for PF-TT, our method achieves good reconstructions with measurements using only a single rotation axis. This means that both experiments and reconstructions can be carried out in a slice-by-slice manner which enables single-slice experiments to be performed faster. This simplifies the experimental procedure by not requiring a second rotation stage, facilitating *in situ* experiments using various existing sample environments at a range of synchrotron endstations.

Our findings suggest that ODF-TT with grid-type basis functions will be able to extend the range of samples that can be characterized with existing 3D-XRD and PF-TT techniques. This includes twinned and deformed metal microstructures and broadly mosaic biominerals.

## Figures and Tables

**Figure 6:**
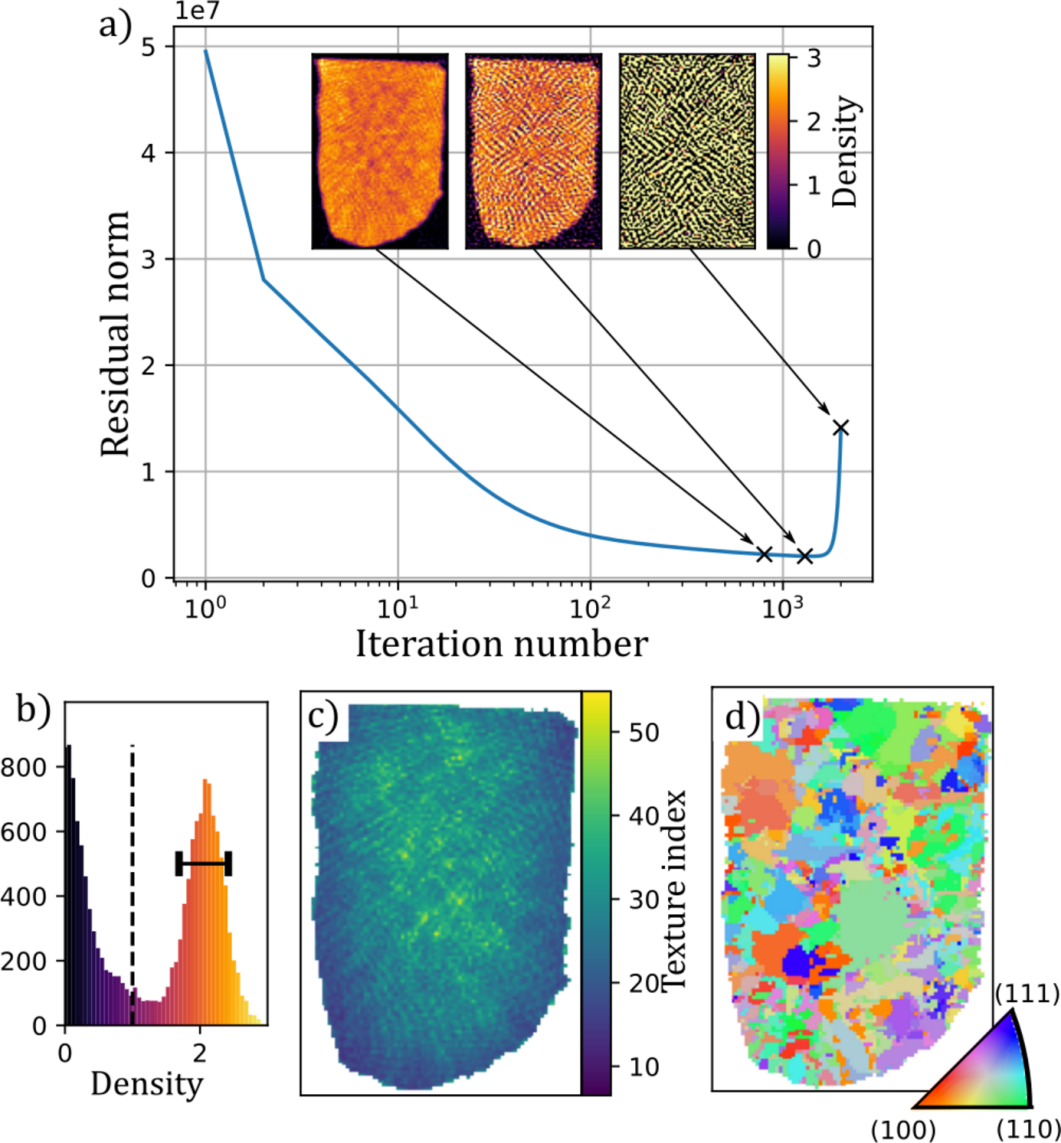
Tomograms of the martensite reconstruction without utilizing the non-negativity constraint. (*a*) shows a convergence curve and the reconstructed density at three different points during the optimization and (*b*) displays a histogram of the density values of the 800th iterate. (*c*) shows the corresponding calculated texture index and (*d*) shows an inverse pole figure map of the main orientation.

**Figure 8:**
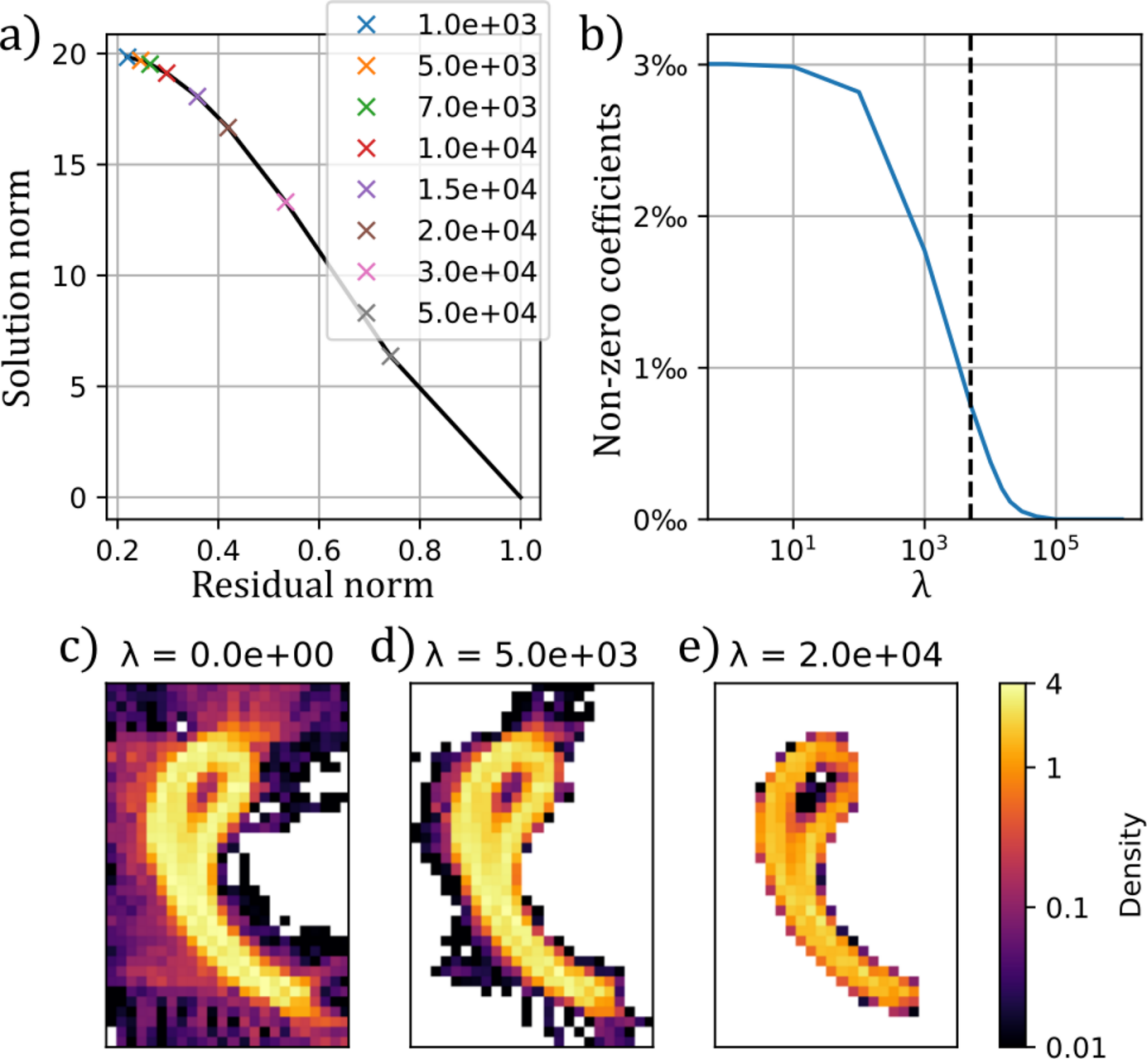
Effect of regularization. (*a*) shows how the residual norm (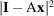) and solution norm (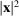) after 500 iterations depend on the choice of the regularization parameter λ. (*b*) Percentage of non-zero coefficients as a function of regularization parameter. At 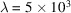 (dashed line) the number of non-zero coefficients has been reduced by about a factor of 4. Plots of the reconstructed density at (*c*) 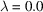, (*d*) 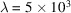 and (*e*) 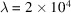. White regions in these images correspond to voxels where all coefficients are zero.

**Figure 9:**
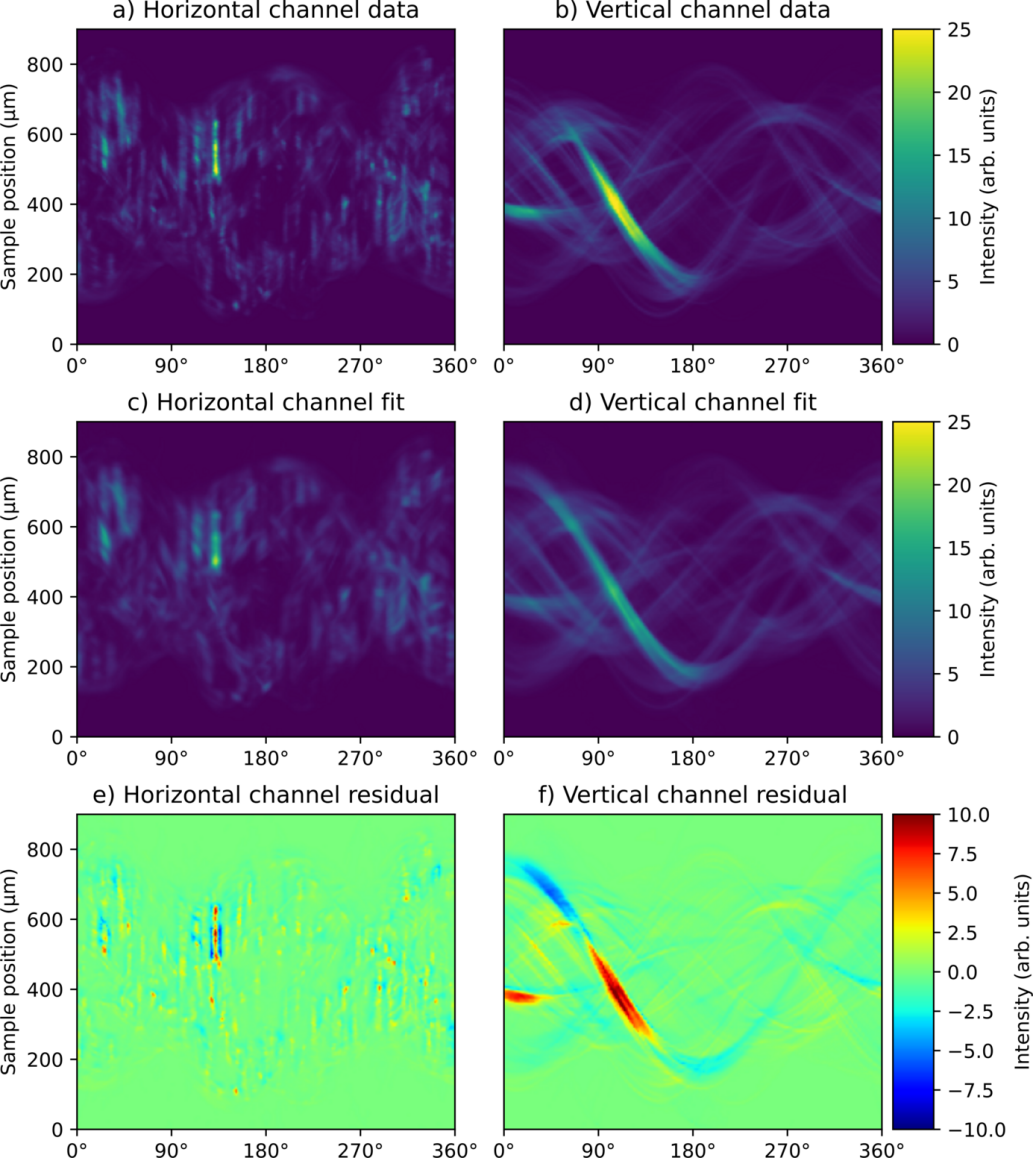
Comparison of data and fit of the martensite reconstruction. (*a*, *b*) Experimental sinograms of the 110 peak for a single azimuthal detector channel. (*c*, *d*) Fitted sinograms of the same angular channel. (*e*, *f*) Residual. (*a*, *c*, *e*) show an angular channel orthogonal to the rotation axis and (*b*, *d*, *f*) show an angular channel parallel to the rotation axis.

## Appendix A

### . Importance of the non-negativity constraint

To illustrate the importance of the non-negativity constraint, we performed a reconstruction of the martensite data without using the non-negativity constraint. Fig. 6 ▸ shows the results of this reconstruction. It is apparent that the reconstruction without the non-negativity constraint suffers from a high-frequency reconstruction artifact that grows exponentially in amplitude after about 1000 iterations. The fixed-step-size algorithm used here is not stable for this problem, but a reasonable reconstruction can be achieved by stopping the optimization procedure early.

### . Comparison of pole figures

To give more insight into the comparison of ODF-TT and PF-TT, we plot the {200} pole figure of a single voxel of the reconstructions of the gastropod shell data in Fig. 7 ▸. It appears that the PF-TT reconstruction does reconstruct significant scattering in the direction where ODF-TT shows the majority of the intensity. However, the PF-TT reconstruction also has significant intensity in two other directions with similar amplitude.

### . L1 regularization

For the samples investigated in this paper, good reconstructions can be achieved without use of L1 regularization. However, there are advantages to using the regularization. As seen in Fig. 8 ▸, using a regularized reconstruction can reduce streaking artifacts in the reconstruction and significantly decrease the number of non-zero coefficients. The latter is useful as it makes the reconstructed volumes easier to store and process for further analysis.

### . Residuals

To get some insight into the quality of the fit and potential source of errors, we show the fit and residuals in Fig. 9 ▸. The magnitude of the residual is lower than 2 throughout most of both sinograms, but peaks at a large value of around ±10 near the most intense features in each sinogram. We believe that these large errors are due to the limited angular resolution of the reconstruction. The two sinograms displayed here were chosen arbitrarily. The full dataset contains 504 such sinograms.
